# High level amplification of N-MYC is not associated with adverse histology or outcome in primary retinoblastoma tumours

**DOI:** 10.1038/sj.bjc.6600532

**Published:** 2002-09-23

**Authors:** D M Lillington, L K Goff, J E Kingston, Z Onadim, E Price, P Domizio, B D Young

**Affiliations:** Cancer Research UK, Department of Medical Oncology, St Bartholomew's and the Royal London NHS Trust, London, EC1M 6BQ, UK; Paediatric Oncology, St Bartholomew's and the Royal London NHS Trust, London, EC1A 7BE, UK; Retinoblastoma Unit, St Bartholomew's and the Royal London NHS Trust, London, EC1A 7BE, UK; Department of Pathology, St Bartholomew's and the Royal London NHS Trust, London, EC1A 7BE, UK; Department of Histopathology, St Bartholomew's and the Royal London NHS Trust, London, EC1A 7BE, UK

**Keywords:** N-MYC, retinoblastoma, real-time quantitative polymerase chain reaction (RQ–PCR), outcome

## Abstract

Twenty-five primary retinoblastoma tumours were analysed by real-time quantitative polymerase chain reaction to determine the genomic copy number of the N-MYC gene (2p24) relative to the copy number for REL, B2M, ALB, AF10 and MLL. Twenty-one of these tumours were shown by Comparative Genomic Hybridization to contain variable copy number increases of chromosomal material mapping to 2p. High level amplification (>30-fold) of N-MYC was found in three tumours, none of which showed adverse histological features and all patients are surviving at between 54 and 108 months post enucleation. Furthermore, the three tumours associated with metastasis and adverse patient outcome showed normal N-MYC copy number. Although high level amplification of N-MYC is an unfavourable prognostic indicator in neuroblastoma, these data show no evidence of a correlation between amplification of N-MYC and adverse outcome in retinoblastoma.

*British Journal of Cancer* (2002) **87**, 779–782. doi:10.1038/sj.bjc.6600532 www.bjcancer.com

© 2002 Cancer Research UK

## 

Retinoblastoma (Rb) is an intraocular malignancy affecting young children with a frequency of one per 20 000 live births (Knudson, 1971). Mutation of the RB1 tumour suppressor gene, located at chromosome 13 band q14, is essential to the pathogenesis of Rb. In hereditary cases (40–45% of cases) there is a germline mutation of RB1, thereby conferring predisposition, and the second allele is inactivated by somatic mutation. This ‘two hit’ mechanism was first hypothesised by Knudson (Knudson, 1976) and later substantiated following cloning of RB1 (Friend *et al*, 1986). In patients with germline mutations, bilateral Rb tumours usually develop and patients have an increased risk of secondary cancers, in particular osteosarcoma and soft tissue sarcoma (Draper *et al*, 1986; Roarty *et al*, 1988). In the sporadic unilateral Rb tumours, both RB1 mutations are required in the same somatic cell to initiate tumour formation. The risk factors for the development of sporadic Rb are not clear but it has been suggested that exposure to human papilloma viruses may play a role (Orjuela *et al*, 2000). A variety of chromosomal abnormalities have been reported in Rb tumours (Benedict *et al*, 1983; Squire *et al*, 1985; Mitelman, 2001), including frequent gains of 6p and 1q, although the impact of these additional events on tumorigenesis and prognosis has not been thoroughly investigated. A correlation between gain of 6p and the most malignant behaviour in Rb has been proposed (Oliveros and Yunis, 1995) and a relationship between N-MYC amplification, higher proliferation index (PI), advanced tumour stage and poor clinical outcome was suggested (Kim *et al*, 1999). It is well recognised in haematological malignancies and solid tumours, that a number of recurrent chromosomal abnormalities have independent prognostic importance (Ferrando and Look, 2000; Misra *et al*, 2000; Mrozek *et al*, 2000; Taylor *et al*, 2000; Mrozek *et al*, 2001) and, in particular, amplification of N-MYC is known to be associated with adverse outcome in childhood neuroblastoma (George *et al*, 2001; Lastowska *et al*, 2001). Retinoblastoma, like neuroblastoma, is thought to be a tumour of neuroectodermal origin and it shares a very similar pattern of metastasis to neuroblastoma, metastasising predominantly to bone, bone marrow, nodes and central nervous system. N-MYC is also a target for amplification in childhood Rb but whether this represents a molecular marker for the assessment of prognosis in this tumour type is not known. Real-time quantitative polymerase chain reaction (RQ–PCR) using the TaqMan system provides a rapid and sensitive method for the analysis of gene dosage (Goff *et al*, 2000; Tajiri *et al*, 2001). There was considerable variation, by CGH, in the extent of gain for 2p23–25 in these Rb tumours and thus RQ–PCR was used to determine N-MYC copy number and this was correlated with tumour histology and outcome.

## MATERIALS AND METHODS

### Tumour samples

Tumour samples were obtained following enucleation and DNA extracted using Nucleon BACC kits (Amersham International plc, UK) according to the manufacturer's protocol. Twenty-one tumour DNA samples were identified by CGH to show gain of 2p material and these were selected for RT–PCR analysis. In addition, tumour DNA from a further four patients, including three (056, 035, 048) from the poor histo-prognostic risk (PR) category two of whom died, was analysed although none had evidence for gain of 2p by CGH. Seven tumours were derived from six bilaterally affected patients and two patients had a positive family history (one bilateral and one unilateral case). The histological risk, clinical details, CGH findings, treatment and outcome are summarised in Tables 1

**Table 1:**
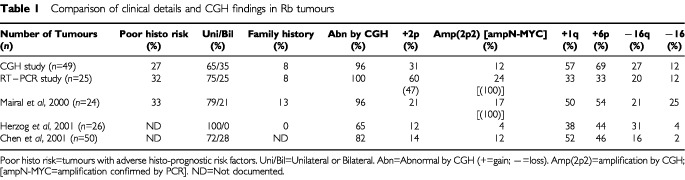
Comparison of clinical details and CGH findings in Rb tumours

and 2

**Table 2:**
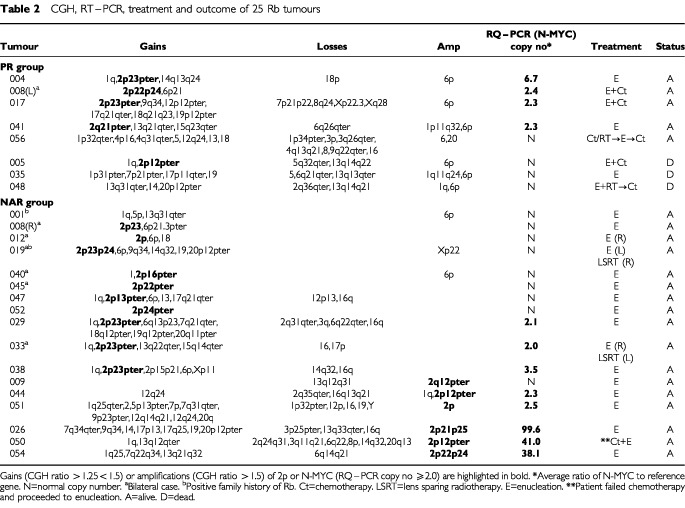
CGH, RT–PCR, treatment and outcome of 25 Rb tumours

. CGH was performed using Vysis CGH kits (Vysis, Inc., France) according to the recommended protocol. The risk factors were assigned according to the criteria of Khelfaoui *et al* (1996) and the study was approved by the East London and The City Health Authority Research Ethics Committee.

## Real-time quantitative polymerase chain reaction(RQ–PCR)

RQ–PCR analysis was performed using the ABI Prism 7700 sequence detector system (Taqman assay). Primers for N-MYC were chosen from intron 2 with the assistance of software Primer Express version 1.0 (ABI/Perkin Elmer, Foster City, CA, USA). The Accession Number for the sequence used was Y00664. The N-MYC primer sequences were 5′–3′ forward GAAGATATGTTTTGATTTTCATGCTTG, used at 300 nmol l^-1^ and reverse TGTAGCATCATGTGCGCATTC, used at 900 nmol l^−1^. The Taqman probe for N-MYC carried a 5′ FAM reporter and a TAMRA quencher (Perkin Elmer, Warrington, UK). N-MYC probe sequence was 5′-(FAM)-ACAATAATTTTCTACCCCAGCGTGGTAGTCAATG-(TAMRA)-3′, used at 100 nmol l^−1^. PCR amplification was performed as previously described (Goff *et al*, 2000). Each assay included ‘no template’ control and standard curves using placental DNA diluted over five logs. To ascertain N-MYC copy number, the copy number of B2M (15q21-22), ALB (4q11-13), MLL (11q23), AF10 (10p12) and REL (2p12-13) (reference genes) was also investigated. For each tumour the ratio of N-MYC copy number/ reference gene copy number was determined and a value above two considered indicative of amplification (Bieche *et al*, 1998). N-MYC copy number was deduced using a minimum of three reference genes selected on the basis of the CGH results being normal for those regions. All standard curves had a correlation coefficient of at least 0.99. The primer oligonucleotides were synthesized by the Imperial Cancer Research Fund, Oligonucleotide Synthesis Service.

## RESULTS AND DISCUSSION

**Figure 1:**
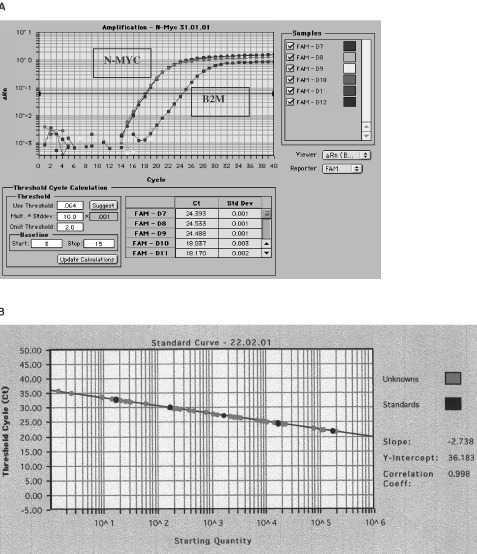
(**A**) RQ–PCR results showing the amplification plot for N-MYC and B2M (tumour 206). N-MYC:B2M copy number ratio=99.6. (**B**) Standard Curve for B2M. Black dots represent standard log dilutions, grey dots represent test DNA. The correlation coefficient for this standard curve was 0.998.

The RQ–PCR results are summarised in Table 2. Three tumours showed greater than 10-fold increase in N-MYC copy number (026, 050 and 054) at 100-, 41- and 38-fold respectively. Figure 1a

represents the amplification plot for N-MYC and B2M from tumour 026 and a typical standard curve (B2M) is shown in Figure 1b. Two of the three patients (026 and 054) were diagnosed at less than 12 months (7 and 3 months, respectively) whilst the third patient (050) presented at 67 months. Contrary to the findings by Mairal *et al* (2000) that tumours with amplification of N-MYC show mild differentiation, all three tumours studied here were poorly differentiated. All three patients are alive at 108 (026), 65 (050) and 54 (054) months post enucleation and although the tumours were poorly differentiated, none of them demonstrated either of the recognised adverse histological features, namely deep choroidal or retrolaminar invasion of the optic nerve. The four tumours reported by Mairal *et al* (2000) with amplification of N-MYC also lacked adverse histoprognostic risk factors thereby strengthening a lack of association of multiple copies of N-MYC with poor outcome. Despite gain of 6p being a frequent CGH finding in Rb (34/49 tumours studied by CGH, data not shown), all three tumours with high level amplification of N-MYC lacked gain of 6p. It has been suggested that gain of 6p is associated with more aggressive tumours (Oliveros and Yunis, 1995) and hence this lends further support to the hypothesis that high N-MYC copy number is not an adverse feature since these tumours also lacked gain of 6p. The three patients who died following extra-ocular relapse (005, 035 and 048) showed no gain of N-MYC by RT–PCR and two also lacked gain of 2p by CGH. Of those tumours with normal N-MYC copy number by RQ–PCR, there were five tumours showing gain of the entire 2p arm by CGH and 4 showing gain of just the 2p2 region. In all cases CGH suggested 0.5-fold increase (i.e. one additional copy) and hence RQ–PCR may not be reliable enough to discern trisomy. The presence of any normal cells in the tumour will also hinder the ability to detect trisomy but this would have been applicable to the CGH experiments too. It is also possible that other genes mapping within 2p2 e.g. Id2 may be the targets for low level gain in this region. Id2 belongs to a family of Id genes exerting an inhibitory effect on transcription by heterodimerising with basic helix–loop–helix proteins. Recently, Lasorella *et al* (2002) demonstrated the correlation of Id2 and N-MYC expression and suggested that Id2 overexpression in neuroblastoma may be a better prognostic indicator than N-MYC amplification. Four tumours showing adverse histology did show gain of N-MYC; in tumours 017, 08L and 041 the increase was between 2–3-fold and in tumour 004 there was a 6.7-fold increase.

In summary, molecular markers have allowed more precise assessment of an individual patient's prognosis in a number of malignant diseases and, in particular, N-MYC copy number is an important prognostic indicator in neuroblastoma and influences management. In the retinoblastomas studied in this series, tumours with amplification of N-MYC copy number (>30-fold) were not associated with adverse histology or outcome, nor were they associated with gain of 6p. Those tumours with adverse histology and adverse outcome (death following metastasis) did not exhibit either low or high copy number gain of N-MYC. The presence of multiple copies of N-MYC in Rb does not, therefore, appear to predict a poor clinical outcome.
